# 1-(3,5-Dinitro­benzo­yl)-3,3-dipropyl­thio­urea

**DOI:** 10.1107/S1600536811013638

**Published:** 2011-04-16

**Authors:** Sohail Saeed, Naghmana Rashid, Muhammad Sher, Seik Weng Ng, Edward R. T. Tiekink

**Affiliations:** a Department of Chemistry, Research Complex, Allama Iqbal Open University, Islamabad 44000, Pakistan; b Department of Chemistry, University of Malaya, 50603 Kuala Lumpur, Malaysia

## Abstract

The title thio­urea derivative, C_14_H_18_N_4_O_5_S, features two substantial twists between its component fragments: the dihedral angle between the SN_2_C (thio­urea) and ONC_2_ (amide) residues is 48.89 (7)° and that between the benzene ring and the amide residue is 30.27 (7)°. In the crystal, mol­ecules are linked by bifurcated N—H⋯(O,S) hydrogen bonds, generating [001] supra­molecular chains.

## Related literature

For the biological activity of thio­urea derivatives, see: Venkatachalam *et al.*, (2004 ▶); Saeed *et al.* (2011 ▶). For related thio­urea structures, see: Gunasekaran *et al.* (2010 ▶); Saeed *et al.* (2010 ▶); Dzulkifli *et al.* (2011 ▶).
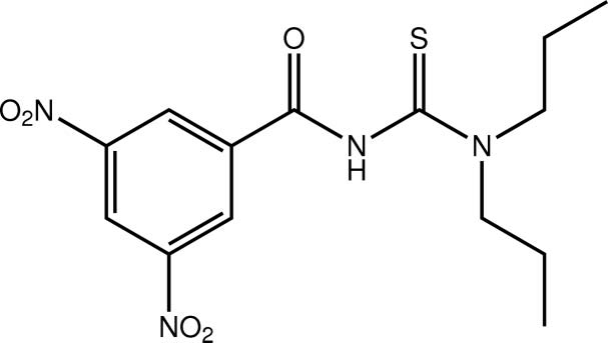


## Experimental

### 

#### Crystal data

C_14_H_18_N_4_O_5_S
*M* *_r_* = 354.38
Monoclinic, 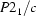

*a* = 7.9406 (4) Å
*b* = 21.2839 (10) Å
*c* = 9.5967 (4) Å
β = 94.379 (4)°
*V* = 1617.17 (13) Å^3^
*Z* = 4
Mo *K*α radiation
μ = 0.23 mm^−1^
*T* = 295 K
0.30 × 0.20 × 0.10 mm

#### Data collection

Agilent SuperNova Dual diffractometer with an Atlas detector
Absorption correction: multi-scan (*CrysAlis PRO*; Agilent, 2010 ▶) *T*_min_ = 0.933, *T*_max_ = 0.977
8055 measured reflections
3614 independent reflections
2878 reflections with *I* > 2σ(*I*)
*R*_int_ = 0.027

#### Refinement

*R*[*F* ^2^ > 2σ(*F* ^2^)] = 0.055
*wR*(*F* ^2^) = 0.154
*S* = 1.02
3614 reflections
221 parameters
1 restraint
H atoms treated by a mixture of independent and constrained refinement
Δρ_max_ = 1.04 e Å^−3^
Δρ_min_ = −0.46 e Å^−3^

### 

Data collection: *CrysAlis PRO* (Agilent, 2010 ▶); cell refinement: *CrysAlis PRO*; data reduction: *CrysAlis PRO*; program(s) used to solve structure: *SHELXS97* (Sheldrick, 2008 ▶); program(s) used to refine structure: *SHELXL97* (Sheldrick, 2008 ▶); molecular graphics: *ORTEP-3* (Farrugia, 1997 ▶) and *DIAMOND* (Brandenburg, 2006 ▶); software used to prepare material for publication: *publCIF* (Westrip, 2010 ▶).

## Supplementary Material

Crystal structure: contains datablocks global, I. DOI: 10.1107/S1600536811013638/hb5845sup1.cif

Structure factors: contains datablocks I. DOI: 10.1107/S1600536811013638/hb5845Isup2.hkl

Additional supplementary materials:  crystallographic information; 3D view; checkCIF report

## Figures and Tables

**Table 1:** Hydrogen-bond geometry (Å, °)

*D*—H⋯*A*	*D*—H	H⋯*A*	*D*⋯*A*	*D*—H⋯*A*
N2—H2⋯O1^i^	0.87 (1)	2.53 (2)	3.264 (3)	142 (2)
N2—H2⋯S1^i^	0.87 (1)	2.69 (2)	3.436 (2)	144 (2)

Symmetry code: (i) 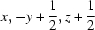.

**Fig. 1.**
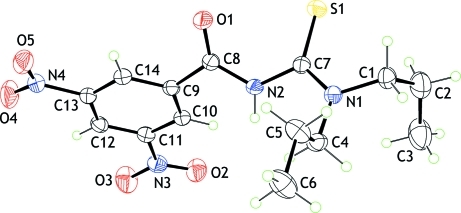
The molecular structure of (I) showing displacement ellipsoids at the 35% probability level.

**Fig. 2.**
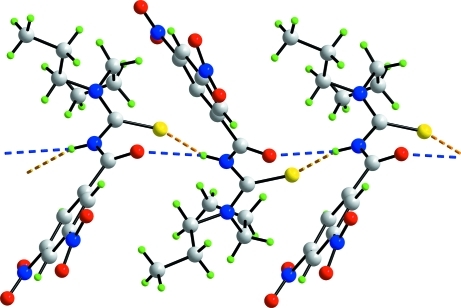
Supramolecular chain aligned along the c axis in (I) mediated by N—H···O, S hydrogen bonding shown as blue and orange dashed lines, respectively.

## supplementary crystallographic information

### Comment

The biological potential of thiourea derivatives (Venkatachalam *et al.*, 2004; Saeed *et al.*, 2011) motivates structural studies of these compounds (Gunasekaran *et al.* 2010; Saeed *et al.* 2010; Dzulkifli *et al.*, 2011). Herein, the crystal and molecular structure of the title thiourea derivative, (I), is described.

The molecular structure of (I), Fig. 1, shows a significant twist around the central atoms as seen in the value of the dihedral angle formed between the least-squares planes through the S1,N1,N2,C7 (thiourea) and O1,N2,C8,C9 (amide) atoms of 48.89 (7) °. Further, the benzene ring is twisted out of the plane of the carbonyl residue as indicated by the O1—C8—C9—C10 torsion angle of 147.1 (2) °. With respect to the S1,N1,N2,C7 plane, the n-propyl groups lie to either side. Whereas the O2-nitro group is co-planar with the benzene ring to which it is bonded, the O2—N3—C11—C10 torsion angle = -4.2 (3) °, the O4-nitro group is slightly twisted out of the plane as seen in the value of the O4—N4—C13—C12 torsion angle of -9.3 (3) °.

The crystal packing is dominated by N—H···O,*S* hydrogen bonds as the N1—H H atoms is bifurcated, Table 1. These result in the formation of six-membered {···H···OCNCS} synthons and linear supramolecular chains along the *c* direction, Fig. 2.

### Experimental

A solution of 3,5-dinitrobenzoyl chloride (0.01 mol) in anhydrous acetone (75 ml) and 3% tetrabutylammonium bromide (TBAB) as a phase-transfer catalyst (PTC) in anhydrous acetone was added drop-wise to a suspension of dry potassium thiocyanate (0.01 mol) in acetone (50 ml) and the reaction mixture was refluxed for 50 min. After cooling to room temperature, a solution of dipropyl amine (0.01 mol) in anhydrous acetone (25 ml) was added drop-wise and the resulting mixture refluxed for 3 h. Hydrochloric acid (0.1 N, 300 ml) was added and the solution was filtered. The solid product was washed with water and purified by re-crystallization from ethyl acetate to yield light-yellow prisms of (I). Yield: 1.29 g (82%); *M*.pt. 407–408 K. IR (KBr, cm^-1^): 3173 ν(NH), 1690 ν(C═O), 1536 ν(benzene ring), 1180 ν(C═S). Anal. Calcd. for C_14_H_18_N_4_O_5_S: C, 47.45; H, 5.12; N, 15.81; S, 9.05%. Found: C, 47.53; H, 5.17; N, 15.75; S, 9.03%.

### Refinement

Carbon-bound H-atoms were placed in calculated positions [C—H 0.93–0.97 Å, *U*_iso_(H) 1.2–1.5*U*_eq_(C)] and were included in the refinement in the riding model approximation. The amino H-atoms were located in a difference Fourier map, and were refined with a distance restraint of N—H 0.88±0.01 Å; the *U*_iso_ values were refined. The maximum and minimum residual electron density peaks of 1.04 and 0.46 e Å^-3^, respectively, were located 1.05 Å and 0.33 Å from the C2 and H2a atoms, respectively.

### Crystal data

**Table:**

C_14_H_18_N_4_O_5_S	*F*(000) = 744
*M**_r_* = 354.38	*D*_x_ = 1.456 Mg m^−^^3^
Monoclinic, *P*2_1_/*c*	Mo *K*α radiation, λ = 0.71073 Å
Hall symbol: -P 2ybc	Cell parameters from 3443 reflections
*a* = 7.9406 (4) Å	θ = 2.3–29.3°
*b* = 21.2839 (10) Å	µ = 0.23 mm^−^^1^
*c* = 9.5967 (4) Å	*T* = 295 K
β = 94.379 (4)°	Prism, light yellow
*V* = 1617.17 (13) Å^3^	0.30 × 0.20 × 0.10 mm
*Z* = 4	

### Data collection

**Table:**

Agilent SuperNova Dual diffractometer with an Atlas detector	3614 independent reflections
Radiation source: SuperNova (Mo) X-ray Source	2878 reflections with *I* > 2σ(*I*)
Mirror	*R*_int_ = 0.027
Detector resolution: 10.4041 pixels mm^-1^	θ_max_ = 27.5°, θ_min_ = 2.3°
ω scans	*h* = −10→10
Absorption correction: multi-scan (*CrysAlis PRO*; Agilent, 2010)	*k* = −27→26
*T*_min_ = 0.933, *T*_max_ = 0.977	*l* = −12→10
8055 measured reflections	

### Refinement

**Table:**

Refinement on *F*^2^	Primary atom site location: structure-invariant direct methods
Least-squares matrix: full	Secondary atom site location: difference Fourier map
*R*[*F*^2^ > 2σ(*F*^2^)] = 0.055	Hydrogen site location: inferred from neighbouring sites
*wR*(*F*^2^) = 0.154	H atoms treated by a mixture of independent and constrained refinement
*S* = 1.02	*w* = 1/[σ^2^(*F*_o_^2^) + (0.0697*P*)^2^ + 1.1729*P*] where *P* = (*F*_o_^2^ + 2*F*_c_^2^)/3
3614 reflections	(Δ/σ)_max_ = 0.001
221 parameters	Δρ_max_ = 1.04 e Å^−^^3^
1 restraint	Δρ_min_ = −0.46 e Å^−^^3^

### Fractional atomic coordinates and isotropic or equivalent isotropic displacement parameters (Å^2^)

**Table:**

	*x*	*y*	*z*	*U*_iso_*/*U*_eq_	
S1	0.37257 (8)	0.21276 (3)	0.24866 (6)	0.0405 (2)	
O1	0.7561 (2)	0.24758 (9)	0.29218 (19)	0.0505 (5)	
O2	0.5844 (3)	0.44242 (11)	0.7375 (2)	0.0621 (6)	
O3	0.8149 (3)	0.49393 (11)	0.7748 (3)	0.0761 (7)	
O4	1.2906 (3)	0.44304 (11)	0.5156 (3)	0.0711 (7)	
O5	1.3052 (3)	0.35161 (11)	0.4221 (2)	0.0623 (6)	
N1	0.4129 (2)	0.15013 (9)	0.4887 (2)	0.0334 (4)	
N2	0.5882 (3)	0.23606 (9)	0.4730 (2)	0.0343 (4)	
H2	0.581 (3)	0.2455 (12)	0.5607 (13)	0.044 (7)*	
N3	0.7311 (3)	0.45149 (10)	0.7190 (2)	0.0451 (5)	
N4	1.2306 (3)	0.39224 (11)	0.4807 (2)	0.0440 (5)	
C1	0.2706 (3)	0.10939 (12)	0.4387 (3)	0.0433 (6)	
H1A	0.2787	0.0701	0.4899	0.052*	
H1B	0.2802	0.0999	0.3408	0.052*	
C2	0.0973 (4)	0.1384 (2)	0.4550 (4)	0.0746 (10)	
H2A	0.0819	0.1733	0.3903	0.089*	
H2B	0.0117	0.1073	0.4280	0.089*	
C3	0.0687 (5)	0.1608 (2)	0.5930 (5)	0.0882 (13)	
H3A	−0.0414	0.1795	0.5919	0.132*	
H3B	0.1528	0.1915	0.6217	0.132*	
H3C	0.0757	0.1262	0.6574	0.132*	
C4	0.5096 (3)	0.12842 (12)	0.6168 (2)	0.0396 (6)	
H4A	0.4329	0.1098	0.6791	0.047*	
H4B	0.5640	0.1641	0.6642	0.047*	
C5	0.6417 (4)	0.08077 (13)	0.5847 (3)	0.0510 (7)	
H5A	0.7223	0.1002	0.5270	0.061*	
H5B	0.5880	0.0464	0.5318	0.061*	
C6	0.7351 (4)	0.05486 (15)	0.7163 (4)	0.0659 (9)	
H6A	0.8211	0.0262	0.6912	0.099*	
H6B	0.6569	0.0332	0.7708	0.099*	
H6C	0.7862	0.0888	0.7701	0.099*	
C7	0.4603 (3)	0.19711 (10)	0.4084 (2)	0.0313 (5)	
C8	0.7150 (3)	0.26338 (11)	0.4063 (2)	0.0339 (5)	
C9	0.8066 (3)	0.31654 (10)	0.4830 (2)	0.0320 (5)	
C10	0.7262 (3)	0.35735 (11)	0.5703 (2)	0.0337 (5)	
H10	0.6152	0.3502	0.5908	0.040*	
C11	0.8147 (3)	0.40847 (11)	0.6256 (2)	0.0350 (5)	
C12	0.9793 (3)	0.42129 (11)	0.5982 (2)	0.0367 (5)	
H12	1.0361	0.4564	0.6352	0.044*	
C13	1.0552 (3)	0.37937 (11)	0.5132 (2)	0.0353 (5)	
C14	0.9729 (3)	0.32754 (11)	0.4549 (2)	0.0338 (5)	
H14	1.0280	0.3004	0.3976	0.041*	

### Atomic displacement parameters (Å^2^)

**Table:**

	*U*^11^	*U*^22^	*U*^33^	*U*^12^	*U*^13^	*U*^23^
S1	0.0449 (4)	0.0445 (4)	0.0315 (3)	−0.0042 (3)	−0.0021 (2)	0.0030 (2)
O1	0.0508 (11)	0.0646 (12)	0.0378 (10)	−0.0172 (9)	0.0146 (8)	−0.0188 (9)
O2	0.0525 (12)	0.0665 (14)	0.0695 (14)	0.0005 (10)	0.0182 (10)	−0.0207 (11)
O3	0.0734 (15)	0.0652 (14)	0.0909 (17)	−0.0130 (12)	0.0146 (13)	−0.0475 (13)
O4	0.0519 (12)	0.0681 (15)	0.0941 (18)	−0.0262 (11)	0.0118 (12)	−0.0156 (13)
O5	0.0438 (11)	0.0814 (15)	0.0636 (13)	−0.0046 (10)	0.0156 (10)	−0.0176 (12)
N1	0.0346 (10)	0.0325 (10)	0.0331 (10)	−0.0015 (8)	0.0025 (8)	−0.0001 (8)
N2	0.0410 (11)	0.0364 (10)	0.0259 (9)	−0.0079 (8)	0.0048 (8)	−0.0052 (8)
N3	0.0514 (14)	0.0432 (12)	0.0409 (12)	0.0023 (10)	0.0037 (10)	−0.0077 (10)
N4	0.0355 (11)	0.0570 (14)	0.0393 (11)	−0.0067 (10)	0.0005 (9)	0.0007 (10)
C1	0.0423 (14)	0.0389 (13)	0.0486 (14)	−0.0094 (11)	0.0036 (11)	−0.0017 (11)
C2	0.0510 (18)	0.097 (3)	0.076 (2)	−0.0187 (18)	0.0090 (16)	−0.015 (2)
C3	0.059 (2)	0.102 (3)	0.107 (3)	−0.015 (2)	0.022 (2)	−0.040 (3)
C4	0.0476 (14)	0.0394 (13)	0.0317 (12)	−0.0022 (11)	0.0031 (10)	0.0047 (10)
C5	0.0528 (16)	0.0482 (15)	0.0500 (16)	0.0068 (13)	−0.0093 (13)	−0.0058 (12)
C6	0.072 (2)	0.0484 (17)	0.072 (2)	0.0038 (15)	−0.0257 (17)	0.0032 (15)
C7	0.0323 (11)	0.0305 (11)	0.0315 (11)	0.0008 (9)	0.0055 (9)	−0.0042 (9)
C8	0.0363 (12)	0.0348 (12)	0.0308 (11)	−0.0036 (9)	0.0035 (9)	−0.0035 (9)
C9	0.0362 (12)	0.0337 (11)	0.0259 (10)	−0.0029 (9)	0.0015 (9)	0.0012 (9)
C10	0.0353 (12)	0.0372 (12)	0.0285 (11)	−0.0025 (10)	0.0019 (9)	0.0023 (9)
C11	0.0398 (13)	0.0344 (12)	0.0304 (11)	0.0023 (10)	0.0006 (9)	−0.0022 (9)
C12	0.0424 (13)	0.0351 (12)	0.0318 (11)	−0.0063 (10)	−0.0024 (10)	−0.0029 (9)
C13	0.0341 (12)	0.0416 (13)	0.0299 (11)	−0.0042 (10)	0.0002 (9)	0.0037 (9)
C14	0.0364 (12)	0.0363 (12)	0.0290 (11)	0.0003 (10)	0.0040 (9)	−0.0005 (9)

### Geometric parameters (Å, °)

**Table:**

S1—C7	1.668 (2)	C3—H3B	0.9600
O1—C8	1.214 (3)	C3—H3C	0.9600
O2—N3	1.207 (3)	C4—C5	1.508 (4)
O3—N3	1.221 (3)	C4—H4A	0.9700
O4—N4	1.218 (3)	C4—H4B	0.9700
O5—N4	1.211 (3)	C5—C6	1.519 (4)
N1—C7	1.334 (3)	C5—H5A	0.9700
N1—C4	1.473 (3)	C5—H5B	0.9700
N1—C1	1.475 (3)	C6—H6A	0.9600
N2—C8	1.364 (3)	C6—H6B	0.9600
N2—C7	1.417 (3)	C6—H6C	0.9600
N2—H2	0.871 (10)	C8—C9	1.507 (3)
N3—C11	1.474 (3)	C9—C14	1.388 (3)
N4—C13	1.476 (3)	C9—C10	1.394 (3)
C1—C2	1.527 (4)	C10—C11	1.379 (3)
C1—H1A	0.9700	C10—H10	0.9300
C1—H1B	0.9700	C11—C12	1.380 (3)
C2—C3	1.441 (5)	C12—C13	1.378 (3)
C2—H2A	0.9700	C12—H12	0.9300
C2—H2B	0.9700	C13—C14	1.379 (3)
C3—H3A	0.9600	C14—H14	0.9300
			
C7—N1—C4	124.44 (19)	C4—C5—C6	112.2 (2)
C7—N1—C1	119.7 (2)	C4—C5—H5A	109.2
C4—N1—C1	115.12 (19)	C6—C5—H5A	109.2
C8—N2—C7	125.03 (19)	C4—C5—H5B	109.2
C8—N2—H2	117.3 (18)	C6—C5—H5B	109.2
C7—N2—H2	117.5 (18)	H5A—C5—H5B	107.9
O2—N3—O3	123.6 (2)	C5—C6—H6A	109.5
O2—N3—C11	118.3 (2)	C5—C6—H6B	109.5
O3—N3—C11	118.1 (2)	H6A—C6—H6B	109.5
O5—N4—O4	124.5 (2)	C5—C6—H6C	109.5
O5—N4—C13	117.9 (2)	H6A—C6—H6C	109.5
O4—N4—C13	117.5 (2)	H6B—C6—H6C	109.5
N1—C1—C2	113.7 (2)	N1—C7—N2	114.2 (2)
N1—C1—H1A	108.8	N1—C7—S1	124.35 (18)
C2—C1—H1A	108.8	N2—C7—S1	121.38 (17)
N1—C1—H1B	108.8	O1—C8—N2	124.3 (2)
C2—C1—H1B	108.8	O1—C8—C9	119.7 (2)
H1A—C1—H1B	107.7	N2—C8—C9	115.93 (19)
C3—C2—C1	115.8 (3)	C14—C9—C10	119.9 (2)
C3—C2—H2A	108.3	C14—C9—C8	117.6 (2)
C1—C2—H2A	108.3	C10—C9—C8	122.3 (2)
C3—C2—H2B	108.3	C11—C10—C9	118.6 (2)
C1—C2—H2B	108.3	C11—C10—H10	120.7
H2A—C2—H2B	107.4	C9—C10—H10	120.7
C2—C3—H3A	109.5	C10—C11—C12	123.0 (2)
C2—C3—H3B	109.5	C10—C11—N3	118.9 (2)
H3A—C3—H3B	109.5	C12—C11—N3	118.1 (2)
C2—C3—H3C	109.5	C13—C12—C11	116.6 (2)
H3A—C3—H3C	109.5	C13—C12—H12	121.7
H3B—C3—H3C	109.5	C11—C12—H12	121.7
N1—C4—C5	111.5 (2)	C12—C13—C14	122.9 (2)
N1—C4—H4A	109.3	C12—C13—N4	117.9 (2)
C5—C4—H4A	109.3	C14—C13—N4	119.2 (2)
N1—C4—H4B	109.3	C13—C14—C9	119.0 (2)
C5—C4—H4B	109.3	C13—C14—H14	120.5
H4A—C4—H4B	108.0	C9—C14—H14	120.5
			
C7—N1—C1—C2	−79.3 (3)	C8—C9—C10—C11	−173.6 (2)
C4—N1—C1—C2	110.2 (3)	C9—C10—C11—C12	0.2 (3)
N1—C1—C2—C3	−52.9 (4)	C9—C10—C11—N3	−179.4 (2)
C7—N1—C4—C5	−86.1 (3)	O2—N3—C11—C10	−4.2 (3)
C1—N1—C4—C5	83.9 (3)	O3—N3—C11—C10	174.9 (2)
N1—C4—C5—C6	−176.4 (2)	O2—N3—C11—C12	176.2 (2)
C4—N1—C7—N2	−15.8 (3)	O3—N3—C11—C12	−4.8 (4)
C1—N1—C7—N2	174.7 (2)	C10—C11—C12—C13	−1.2 (3)
C4—N1—C7—S1	167.53 (18)	N3—C11—C12—C13	178.5 (2)
C1—N1—C7—S1	−2.0 (3)	C11—C12—C13—C14	1.2 (3)
C8—N2—C7—N1	144.3 (2)	C11—C12—C13—N4	179.3 (2)
C8—N2—C7—S1	−38.9 (3)	O5—N4—C13—C12	170.3 (2)
C7—N2—C8—O1	−16.5 (4)	O4—N4—C13—C12	−9.3 (3)
C7—N2—C8—C9	163.4 (2)	O5—N4—C13—C14	−11.5 (3)
O1—C8—C9—C14	−27.4 (3)	O4—N4—C13—C14	168.9 (2)
N2—C8—C9—C14	152.7 (2)	C12—C13—C14—C9	−0.3 (3)
O1—C8—C9—C10	147.1 (2)	N4—C13—C14—C9	−178.4 (2)
N2—C8—C9—C10	−32.8 (3)	C10—C9—C14—C13	−0.7 (3)
C14—C9—C10—C11	0.7 (3)	C8—C9—C14—C13	173.9 (2)

### Hydrogen-bond geometry (Å, °)

**Table:**

*D*—H···*A*	*D*—H	H···*A*	*D*···*A*	*D*—H···*A*
N2—H2···O1^i^	0.87 (1)	2.53 (2)	3.264 (3)	142 (2)
N2—H2···S1^i^	0.87 (1)	2.69 (2)	3.436 (2)	144 (2)

Symmetry codes: (i) *x*, −*y*+1/2, *z*+1/2.
